# Local Allergic Rhinitis in Pediatric Patients: Is IgE Dosage in Nasal Lavage Fluid a Useful Diagnostic Method in Children?

**DOI:** 10.22088/acadpub.BUMS.6.3.174

**Published:** 2017-09-26

**Authors:** Laura Colavita, Natalia Catalano, Giovanna Sposito, Saverio Loddo, Bruno Galletti, Carmelo Salpietro, Francesco Galletti, Caterina Cuppari

**Affiliations:** 1 *Department of Pediatrics, Hospital “Umberto I” of Siracusa, Siracusa, Italy.*; 2 *Department of Otolaryngology, University Hospital of Messina, Messina, Italy.*; 3 *Department of Laboratory Diagnostics, Unit of Clinical Pathology, University Hospital of Messina, Messina, Italy.*; 4 *Department of Adult and Childhood Human Pathology, Unit of Genetics and Pediatric Immunology, University Hospital of Messina, Messina, Italy.*

**Keywords:** Local allergic rhinitis, nasal lavage fluid, IgE, children, Non-allergic rhinitis with eosinophilia syndrome (NARES)

## Abstract

Local Allergic Rhinitis (LAR) is an emerging disease. However, its incidence in the pediatric popolution has not yet been studied. The gold standard for the diagnosis is the nasal provocation test that is not everywhere avalaible and difficult to apply in children. The aim of our study was to evaluate the nasal lavage fluid IgE as a biomarker of LAR in children. 54 pediatric patients [IQR 4.0-12.0 years] were divided into 3 groups: study group (26 children with rhinitis symptoms and without evidence of systemic atopy); allergic rhinitis (AR) group (15 children) and 13 healty controls (HC). Every child was subjected to nasal lavage using 2 ml/nostril of physiologic saline solution, that was therefore analyzed by ImmunoCAP to obtain the IgE concentration. Rhinofibroscopy and nasal cytology were performed. Our data showed the presence of higher value of nasal lavage fluid IgE (average of 6.005 UI/ml; range: 4.47-7.74 UI/ml) in 16 out of 26 patients of the study group who therefore may be classified as affected by LAR. We observed a statistically significant difference (P< 0.0001) between NAR/HC group and LAR group, identifying a cut-off of 3.85 UI/ml. Finally, we found a better response to previous AR therapy in the LAR group than in the NAR group. Our data showed the high incidence of LAR in pediatric patients previously classified as NAR. The measurment of IgE in nasal lavage fluid may be considered an easy and rapid method for the diagnosis of LAR in children. Besides, our data add confirmatory evidence about the good response of LAR children to the classic AR therapy.

A high number of children have symptoms of rhinitis without positivity of skin prick test (SPT) or serum sIgE (RAST) to inhlant allergens. Noninfectious rhinitis are traditionally classified as allergic (AR) and non-allergic (NAR), according to the immunological mechanisms. AR is the most common type of rhinitis. Exact data about the real prevalence of NAR are still absent because only few studies have been performed with standardized methods. NARs may be classified into occupational (irritant), drug or food induced, hormonal, emotio-nal, atrophic, non-allergic rhinitis with eosinophilia syndrome (NARES), and idiopathic (1).

Nasal cytology is a useful diagnostic method because it allows the evaluation of different types of inflammation (viral, bacterial, fungal or parasitic), and the identification of same new pathological entities such as NARES , non-allergic rhinitis with mast cells (NARMA), nonallergic rhinitis with neutrophils (NARNE), and non-allergic rhinitis with eosinophils and mast cells (NARESMA). Between these, the NARES is the most common finding, and is characterized by mucosal eosinophilia with good response to nasal steroid (2).

The first evidence of local allergic rhinitis (LAR) came between 1975 and 1979 when researchers demonstrated the presence of IgE in the nasal mucosa of patients affected by rhinitis with and without evidence of systemic atopy. Therefore, a local IgE production could be restricted to nasal mucosa and the SPT response could not be the representative of a local allergic inflammation.

Diagnosis of LAR is based on IgE detection in nasal lavage fluid, a positive nasal provocation test (NAPT) or both in the absence of systemic atopy (negative SPT and/or serum sIgE) (1). The estimated prevalence might reach 25.7% of all rhinitis patients and more than 47% of patients were previously diagnosed with NAR. Besides, the rhinitis symptom onset in childhood was observed in over 36% of LAR subjects (3).

Only few studies have been performed on LAR and in populations exclusively or mainly composed of adults.

The aim of our study was to evaluate the prevalence of LAR in pediatric patients through the non-invasive and non-time-consuming technique of total IgE dosage in nasal lavage fluid, that is particularly useful in children (even in those uncooperative).

## Patients and methods

**Patients**

The study included 56 pediatric patients afferent to the Pediatric Immuno-Allergology Department of the University Hospital of Messina. They were aged between 4 and 12 years (median age 6.4 years). 28 children formed the study group because rhinitis symptoms with negative SPT; 15 children were classified as affected by allergic rhinitis with positive SPT; 13 children were choo-sen as healty controls. The subjects of the study group had to fulfill the following inclusion criteria: presence of rhinitis symptoms (nasal obstruction, rhinorrea, sneezing, itching and/or postnasal drip); negative SPT and serum sIgE. Exclusion criteria were: presence of respiratory infection during the previous 4 weeks; therapy with nasal or oral corti-costeroids, antihistamines or leukotriene modifiers (Montelukast) during the previous 2 weeks.

For every child in the study group, we evaluated the severity of rhinitis, using ARIA classification that identifies intermittent and persistent rhinitis in relation to the duration of symptoms; mild and moderate/severe rhinitis in relation to the severity of symptoms. We defined intermittent a rhinitis with symptoms present for less than 4 days per week and less than 4 weeks; while itwas considered as persistent if the symptoms were present for more than 4 days per week and more than 4 weeks. The division into mild and moderate/severe rhinitis was done by evaluating sleep quality, limitations of daily activities, reduction of school performance through questionnaires; the degree of the child’s discomfort was related to nasal symptoms, using VAS scale (visual analogue scale) or numeric scale. We thus divided the patients into 4 groups: 1) intermittent-mild rhinitis; 2) intermittent-moderate/severe rhinitis; 3) persistent-mild rhinitis; 4) persistent-moderate/severe rhinitis. The Local Istitutional Review Board approved the study, and a written informed consent was obtained from the parents of each child who participated in the study.

**Clinical and laboratory evaluation**

After clinical evaluation and SPT execution, every child enrolled in the study group was subjected to nasal lavage using 2 ml/ nostril of physiologic saline solution (0.9% NaCl). The nasal lavage fluid was therefore analyzed by ImmunoCAP (Thermo Fisher Scientific, Uppsala, Sweden) to obtain the IgE concentration (nasal IgE). The presence of adenoid hypertrophy was evaluated through rhino-fibroscopy. In every child of the study group, 3 nasal mucosa samples for nasal cytology were collected by scraping the middle portion of the inferior turbinate using the Rhinoprobe (Arlington Scientific, Springville, UT, USA).

**Statistical analysis**

The statistical analyses were performed using GraphPad Prism software package (Version 6, GraphPad Software Inc., San Diego, CA, USA), and the results were considered stastically significant at a p-value less than 0.001. Logistic regression analysis and ROC curve were used to evaluate the laboratory findings.

## Results

General clinical and epidemiologic data are shown in Table 1. Of the 56 samples of nasal lavage fluid obtained, IgE concentration was successfully measured in 54 patients. Only in 2 samples of the study group, the measurement was technically impossible due to excessively high viscosity. Therefore the study group was reduced to 26 children.

**Table 1:** Clinical data of the children enrolled in the study.

**Groups**	**Study group**	**AR Patients**	**Healthy Controls**
Subjects (N)	26	15	13
Age (years)	4-12 (median age 6.4)		
Sex (males N)	14	7	6
Groups of rhinitis symptoms severity (N)			
Group 1	6	5	-
Group 2	10	3	-
Group 3	4	4	-
Group 4	6	3	-
Comrbidity (N)			
Asthma	-	2	-
Persistent dry cough and/or infective bronchospasm	10	4	-
Conjunctivitis	8	6	-
Atopic dermatitis	1		-
Food allergy	-		-
Family history of allergy (N)			
Yes	8	2	1
No	5	2	4

Groups of rhinitis symptoms severity (ARIA classification): 1) intermittent-mild rhinitis; 2) intermittent- moderate/ severe rhinitis; 3) persistent-mild rhinitis; 4) persistent-moderate/ severe rhinitis.

**Fig. 1:**
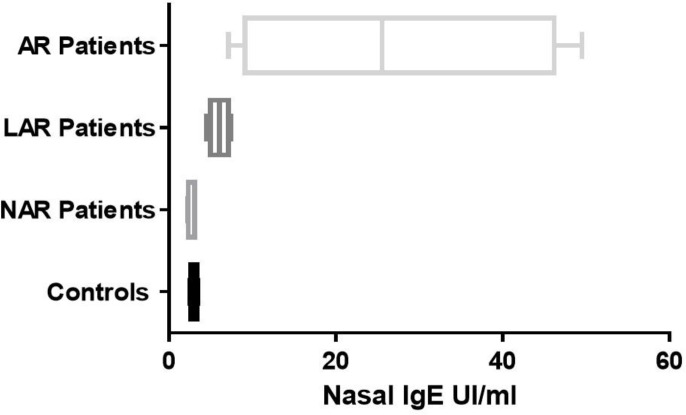
Nasal lavage fluid IgE in the 4 groups of patients. AR: allergic rhinitis; LAR: local allergic rhinitis; NAR: non allergic rhinitis.

**Fig. 2:**
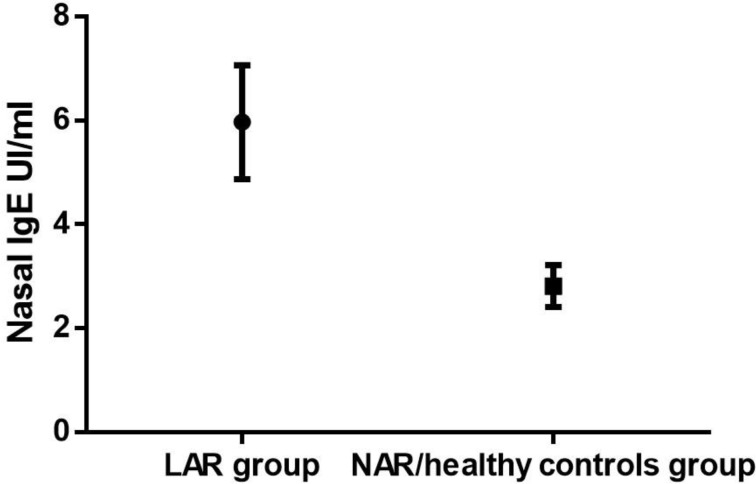
ROC curve data about the IgE concentration in nasal lavage fluid of the LAR patients vs NAR /healthy controls group.

Regarding IgE concentration in nasal lavage fluid, in the control group, we found values between 2.54 and 3.48 UI/ml; in the AR group we found much higher values between 7.16 and 49.5 UI/ml. For the similarity of values of nasal lavage fluid IgE between same patients of the study group and the healthy controls (nasal IgE<3.48 UI/ml), we divided the study group into 2 subgroups: LAR (16 children) and NAR (10 children). LAR children had values between 4.47 and 7.74 UI/ml; NAR patients between 2.22 and 3.07 UI/ml. Therefore, 4 groups of children: LAR, NAR, AR and healthy controls were identified. The determination of the nasal lavage fluid IgE showed stastically significant differences between these 4 groups (P<0.0001) (Figure 1).

**Table 2:** IgE in Nasal Lavage Fluid in LAR patients and in NAR/healthy controls group.

LAR group (Nasal IgE, UI/ml)	NAR/ healthy control groups (Nasal IgE, UI/ml)
7.44	3.04
6.74	2.22
7.27	2.42
5.84	3.07
6.29	3.05
5.09	3.31
4.90	3.04
4.47	2.66
7.32	3.48
6.85	2.54
7.12	3.01
5.53	2.30
6.41	2.23
5.24	3.02
4.77	3.06
4.23	3.01
	2.75
	3.42
	2.33
	2.50
	3.02
	2.27
	3.05

**Fig. 3:**
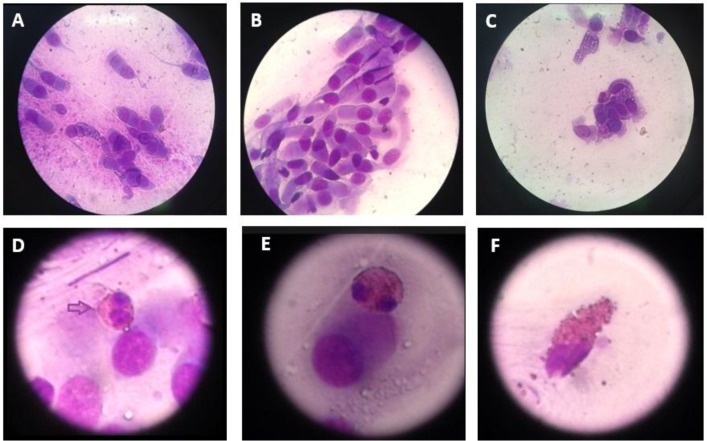
Nasal cytology. A and B: normal rhinocytogram that shows the ciliated pseudostratified epithelium; C, D and E: rhinocytogram of NARES patients; F: partially degranulating eosinophil in NARES rhinocytogram.

**Table 3:** Clinical and laboratory data of the study group, divided into twosubgroups: LAR (local allergic rhinitis) and NAR (non allergic rhinitis).

	LAR patients	NAR patients
Subjects (N)	16	10
Groups of rhinitis symptoms severity (N)		
Group 1	2	4
Group 2	8	2
Group 3	2	2
Group 4	4	2
Family history of allergy (N):		
Yes	10	6
No	6	2
Nasal lavage fluid IgE	6.005+/- 0.3947	2.883+/- 0.1279
NARES cases (N)	2	2
Adenoid hypertrophy≥ 2nd grade (N)	10	6
Response to pregress rhinitis therapy (good response, N)		
Nasal steroid	-	-
Antihistamine	2	2
Montelukast	-	2
Nasal steroid+ Antihistamine	4	2
Antihistamine+ Montelukast	2	-
Montelukast+ Nasal steroid	-	-
Nasal steroid+ Antihistamine+ Montelukast	2	-
Never done therapy	4	2

To find a cutoff, we chose to use only 2 groups, unifying the healthy controls to the NAR children. The difference between the 2 groups (LAR and NAR/healthy controls) relative to IgE concentrations in nasal lavage fluid was stastically significant (P<0.0001) (Figure 2 and Table 2). The best cutoff value of the IgE in nasal lavage fluid was 3.85 UI/ml. We did not find a statistically significant association between the severity of rhinitis symptoms and the nasal lavage fluid IgE concetration (P= 0.9368). In the study group (LAR +NAR), we found only 4 cases of NARES through nasal cytology: 2 in the LAR group and 2 in the NAR group (Figure 3). Only 2 patients of the NAR group were not subjected to rhino- fibroscopy for poor compliance. 16 children out of 24 had adenoid hypertrophy≥ 2nd grade

Finally, we evaluated the response to the eventual rhinitis therapy performed in the past, and we found a better response to treatment with Montelukast, antihistamines and/ or nasal steroids in the LAR group than in the NAR group (Table 3).

## Discussion

Our data show the presence of higher value of nasal lavage fluid IgE in 16 out of 26 pediatric patients with rhinitic symptoms but without signs (positive SPT or serum sIgE) of systemic atopy, who may therefore be classified as affected by LAR. Therefore, the IgE concentration in nasal lavage fluid may be considered as a marker of LAR with a cut off of 3.85 UI/ml for nasal lavage performed with 2 ml /nostril of physiologic saline solution (0.9% NaCl).

Our study did not show a statistically significant association between the severity of rhinitic symptoms and the nasal lavage fluid IgE concetration (P=0.9368). However, a recent study of Occasi et al. showed that symptoms of nasal obstruction were underestimated in children between 6 and 9 years of age when compared with rhinomanometry results (4). In our study we could not perform rhinomanometry; so the real nasal patency, could not be evaluated.

In agreement with the literature data, a high incidence of NARES did not emerge in children with (“overlapped” rhinitis) and without LAR in the present study.

The first evidence of a local sIgE production in nasal mucosa of patients with rhinitis symptoms but without positive SPT came in 1975 by Huggins and Brostoff. They demonstrated that these patients were clinically allergic by NAPT with house-dust-mite, and detected sIgE in their nasal secretions, despite the lack of sIgE in their serum (5).

Other studies have demonstrated a local IgE production in nasal mucosa. Nonetheless, the general mechanism of this is partially understood, involves local-IgE-secreting-B-cells and plasma cells, and the switch of IgG-expressing-B-cells into IgE-expressing-cells (3).

In the nasal mucosa, the lymphoid cells are organized to form the “nasal-associated lymphoid tissue”, and in the intestinal mucosa, the same cells form the GALT (gut- associated lymphoid tissue). T-cells and mast cells produce and release inflammatory mediators (such as IL4, IL13 and CD40L) and so induce locally the B cells switching to IgE producing-plasma cells (6). This type of switching was classically considered as a process localized only in limphoid tissues. Recently this concept has been modified by the finding of epsilon germline transcripts (involved in the B cells switching) and S epsilon S mu DNA switch circles (products of class switch recombination) in nasal mucosal tissue after *ex vivo* allergen challenge, and within lung and sinus of individuals with asthma and sinusitis (7). Confirmatory evidence came from Takha et al. who found mRNA for activation-induced cytidine, multiple germline gene transcripts, and epsilon circle transcripts in the nasal mucosa of allergic patients, indicating a local class switch recombination (8). Another study has demonstrated a local differentation of eosinophils using *in vitro* explant model of allergic nasal mucosa, regulated *in vivo* by endogenous production of sIL-5Ralpha (9). Therefore, IgE is produced directly in the nasal mucosa and cannot be considered the product of migration from regional lymphoid tissue or blood to the nasal mucosa.

To date, only few studies have been done to evaluate the IgE concetration in nasal lavage fluid as a diagnostic marker of LAR, and all of them were performed on populations composed exclusively or mainly of adults.

For example, Rondòn et al. conducted a study in 2007 to evaluate sIgE only to dermatophagoides pt. (DP) and the response to the NAPT with DP in 50 adult patients with persistent nonallergic rhinitis. They used 10 ml of physiological saline for nasal lavage and found increased values of nasal sIgE for DP (ranging from 0.54 to 1.60 UI/ml) in 22% of patients. The possibility that the remaining patients could have specific IgE antibodies to other perennial allergens is a limit of the study (10).

A similar study was conducted on 32 adult patients with seasonal idiopathic rhinitis: in 7/32 cases (21.8%) nasal sIgE ranging from 0.64, to 1.42 kU/l was found, and in all cases nasal total IgE was lower than 18 IU/ml (11).

The diagnosis of seasonal LAR may be done also out of Spring using the nasal allergen provocation test (NAPT). Studies on response to NAPT on LAR patients show 6 peaks of nasal release of sIgE-DP after allergen exposure: immediate and after 6 and 24 h (12, 13) and there is evidence of a fast decrease after avoidance and increase after re-exposure (14).

Other studies have demonstrated the presence of a Th2 inflammatory pattern in nasal mucosa of NAR patients using the same type of sample (nasal secretions) (15) or different samples such as whole, full-thickness nasal turbinate specimens (16, 17). Unfortunately, not all authors have disclosed these same data such as Blom et al. and van Rijswijk et al. who studied nasal biopsy samples (18, 19). These different results may be explained by the existence of more types of NAR that include rhinitis with non IgE-mediated inflammation or characterized by neurogenic mechanisms (such as idiopathic or vasomotor rhinitis) (6).

To date, the diagnosis of LAR is based on the response to NAPT and/or the demonstration of local synthesis of IgE in nasal mucosa. The NAPT is considered the gold-standard in the diagnosis of both LAR and AR. (3). However, this test is not avalaible in all hospitals, time-consuming and difficult to apply in children, especially in younger and uncooperative patients. For this, we have choosen the nasal lavage fluid sample, that is fast and easy to obtain even in younger and uncooperative children. The amount of saline solution (2 ml/nostril of 0.9% NaCl) to be administered for the execution of the nasal wash is less than what is used in the previously mentioned studies conducted on adults (5 ml/nostril). This choice allows the applicability and good tolerance of nasal washes even in younger and uncooperative children and reduces the dilution of the sample, increasing the sensitivity. We believe that the measurement of IgE in nasal lavage fluid is an easy, rapid and really useful diagnostic technique for an early diagnosis of LAR in children, with high specificiy, and acceptable sensitivity.

The importance of an early diagnosis is given by the possibility to start a rhinitis therapy and an allergologic follow-up. Indeed, our data show a good response to classic rhinitis therapy with nasal steroid and/or antihistamine and/or Montelukast in LAR children, in agreement with recent data of literature (3, 10, 11, 20). Therefore, the therapy of LAR and AR should be the same. A recent pilot study on 20 adult patients has given the first evidence of efficacy of specific subcutaneous immunotherapy (SCIT) in patients with LAR sensitized to grass pollen. This study showed a significantly improvement in nasal tolerance to NAPTs and in the clinical response to the natural exposure to aeroallergen after 6 months of preseasonal therapy (21).

Relative to the follow-up and the natural history of LAR, in a recent study on adult LAR patients, Rondòn et al. showed a similar rate of development of systemic atopy compared to healthy controls. This suggests that LAR and classic AR may be 2 independent entities. However, LAR cannot be considered a minor disease because of the significant impairment in health and quality of life of affected patients with a progressive increase in the persistence and severity of nasal symptoms over the years, and the possible association with conjunctivitis and asthma (20).

Our data show the high incidence of LAR, in pediatric patients previously classified as NAR. For all we know, this is the first study conducted only in pediatric patients. The measurment of IgE in nasal lavage fluid may be considered an easy and rapid method to individuate LAR patients in pediatric population, also in younger and uncooperative children, using 2 ml/nostril of saline solution (0.9% NaCL) to perform the nasal wash and considering a cutoff value of 3.85 UI/ml. Besides, our data add confirmatory evidence about the good response of LAR children to the classic AR therapy. To conclude, for the high incidence of LAR and the possibility of NARES in NAR pediatric patients, we believe that it is reasonable to perform a therapeutic trial with the classic AR therapy in NAR children even when the dose of nasal IgE or the NAPT are not executable.
